# The Analogy between the Legal and Nursing Professions

**Published:** 1916-03-04

**Authors:** Charles Russell

**Affiliations:** Bart.


### THE MATRONS' AND SISTERS' DEPARTMENT.

### THE ANALOGY BETWEEN THE LEGAL AND NURSING PROFESSIONS.

By the Hon. SIR CHARLES RUSSELL, Bart.

Being a Speech to the Nursing Profession on the Honourable Arthur Stanley's Scheme for

A COLLEGE OF NURSING.

Really the analogy between the history of solicitors in this country and that of nurses is remarkably close. A hundred years ago there were, to use the expression of one of the speakers, a hundred different doors by which you could become a solicitor. There was what was called the Inns of Chancery, and other teaching schools, through which the candidate could pass. There was no qualifying examination. All that was necessary was that they should have served a certain number of years, and they were entitled to call themselves not, in those days, solicitors, but attorneys. Well, the consequence of that was that the profession fell into disrepute, all social standing was wanting, and the attorney of the farce and the comedy was the buffoon and butt in every play. You can see that if you read some of the old comedies. And just in the same way, of course, in later works of fiction, we get Sarah Gamp and Betsy Prig held up as objects of contempt. Well, we (the solicitors) were in the same position as you are to-day, and we had to struggle, as you are struggling, in order to get out of that position. And we were faced with just the same difficulties as you are faced with. We had a multitude of different little organisations representing our interests, and they all considered that they were "it." Every one of them said that they were the persons who should really lead. And it was only at last, when they formed a voluntary Society, as is proposed in this case, that they got together. And the curious thing about it is that that Society has remained to this day a voluntary Society. The Law Society gradually grew into what is called the Incorporated Law Society, and to this day no solicitor is bound to be a member of it unless he likes, but it was started as a voluntary Society, and has attracted into its body the great majority of the members of the profession—they have become members of the Incorporated Law Society, they have votes, and the Council is elected just as in Mr. Stanley's scheme for a College of Nursing.

Now you will say, How has that led to the State recognition of attorneys and how has that improved the social status? It has led to those results in this way. The first thing our Society did was to take up education; they had lectures, they had examinations, they would not allow people to become members unless they passed those examinations, and at last, after the lapse of some few years, the State handed over to them the entire work of examining solicitors throughout the country, and made it obligatory to pass an examination in general education, and then an examination in matters of law. Time went on, and still further powers were entrusted to the Incorporated Law Society. The entire custody of the Roll was entrusted to them, and instead of moving in the Courts to take people off the Roll, as was done in the old days, this voluntary Society now are the custodians of that Roll, and if you want to remove a solicitor for misconduct or otherwise from the Roll, the application is made to the Incorporated Law Society to investigate the matter and report to the Court, and in nine hundred and ninety-nine cases out of a thousand their report is adopted. There again that voluntary Society, as it is to this day, has been entrusted with these great powers—the entire education of solicitors, their examination and admission, the guardianship of the Roll, and they have actually now, as I have said, disciplinary powers of a very severe order. And yet not one member of the body, curiously enough, is bound to belong to it. It is not compulsory.

So that in the history of the legal profession there is a very marked similarity. The troubles they had are very like the troubles nurses have to-day. We at the beginning had no examination. You have no examination. The consequence was, of course, that there was a low standard of education and a tendency for the legal profession to fall into some disrepute. Immediately we began to insist upon a higher standard of education, immediately we began to raise the standard of legal education, we began to inspire confidence. And remember this—that Parliament is always very prone to help those who help themselves, and if a profession can only get together and decide amongst themselves what they want, then it is a comparatively easy matter to get the State's sanction. But all the time the nurses are waiting for the State to do what no doubt it ought to do, they are running on the wrong lines. I feel that if only the nursing profession can get together, forget any differences which exist, and coalesce so as to form a College of Nursing which will take up the question of education and bring the standard up, it will not be long before Parliament gives to them powers as great as those which have been given to the Incorporated Law Society.

